# Supplementary material for: Analysing human mobility patterns of hiking activities through complex network theory
Source: PLoS One. 2017 May 24;12(5):e0177712. doi: 10.1371/journal.pone.0177712 (PMC5443505; doi:10.1371/journal.pone.0177712)
Supplement: S1 File — (PDF) [file pone.0177712.s001.pdf]

Analysing Human Mobility Patterns of Hiking Activities through Complex Network Theory

Isaac Lera[1*], Toni Pérez[2], Carlos Guerrero[1], Víctor M. Eguíluz[2], Carlos Juiz[1]

1 Departamento de Matemáticas e Informática. Universitat de les Illes Balears, Palma de Mallorca, Spain
2 Instituto de Física Interdisciplinar y Sistemas Complejos IFISC (CSIC-UIB), Palma de Mallorca, Spain

* E-mail: isaac.lera@uib.es (IL)

Supporting Information

Data Description

Our dataset is defined by the activity performed by 2965 users over the last 8 years (from 2009 to 2016) on the Balearic Islands (Spain). This dataset is obtained from a sport tracker application where hikers uploaded the GPX-traces to the platform with the finality of registering and sharing their activity. They used different GPS devices under multiples signal conditions and samplings. As a general description of the dataset, we present the following aggregated metrics: the hiking activity frequency (Figure 1), the time elapsed and the distance travelled during two consecutive samplings (Figure 2 and Figure 3, respectively).

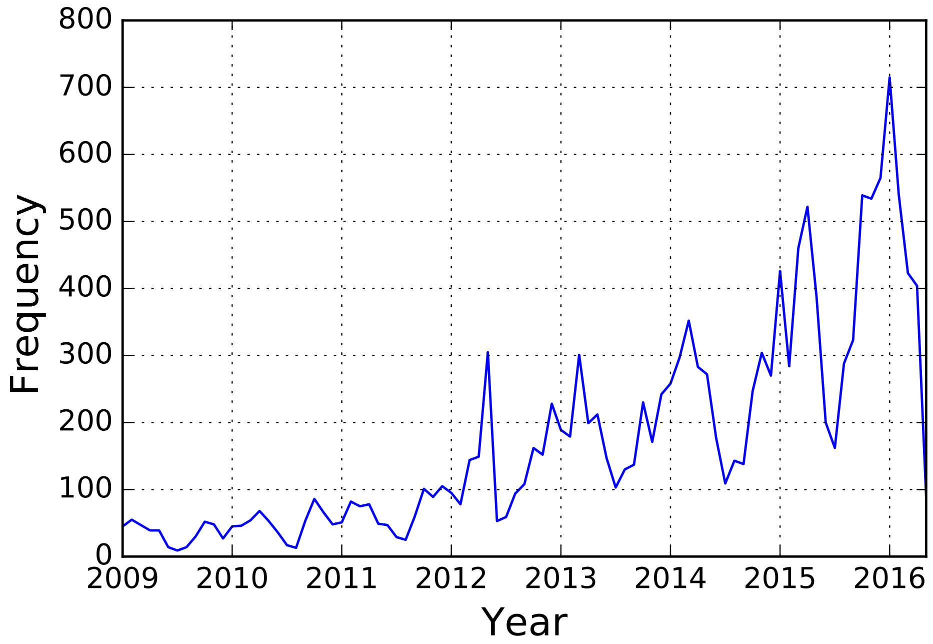

Figure 1. Hiking activity. Histogram of the hiking activity performed during 2009 and 2016.

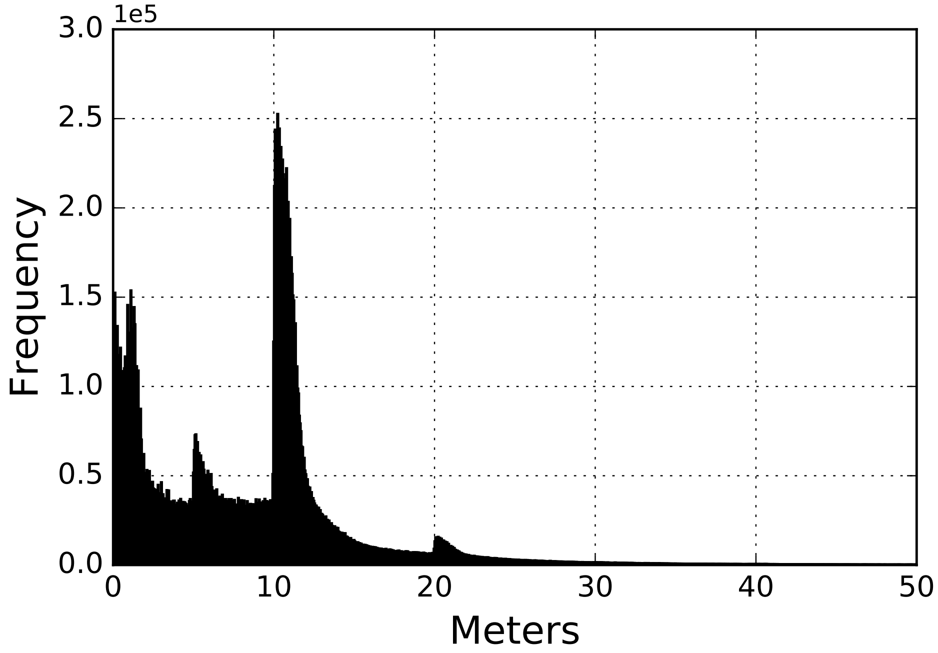

Figure 2. Distance between consecutive records. Histogram of the distance between two consecutive records of the tracks.

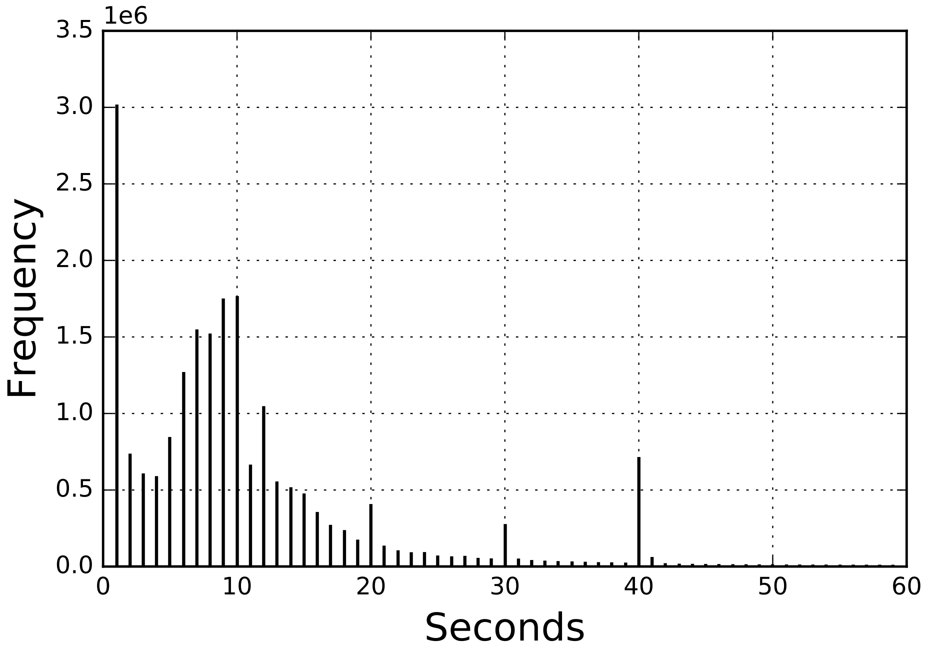

Figure 3. Time between consecutive records. Histogram of the elapsed time between two successive records in the tracks.

Hiking activity networks

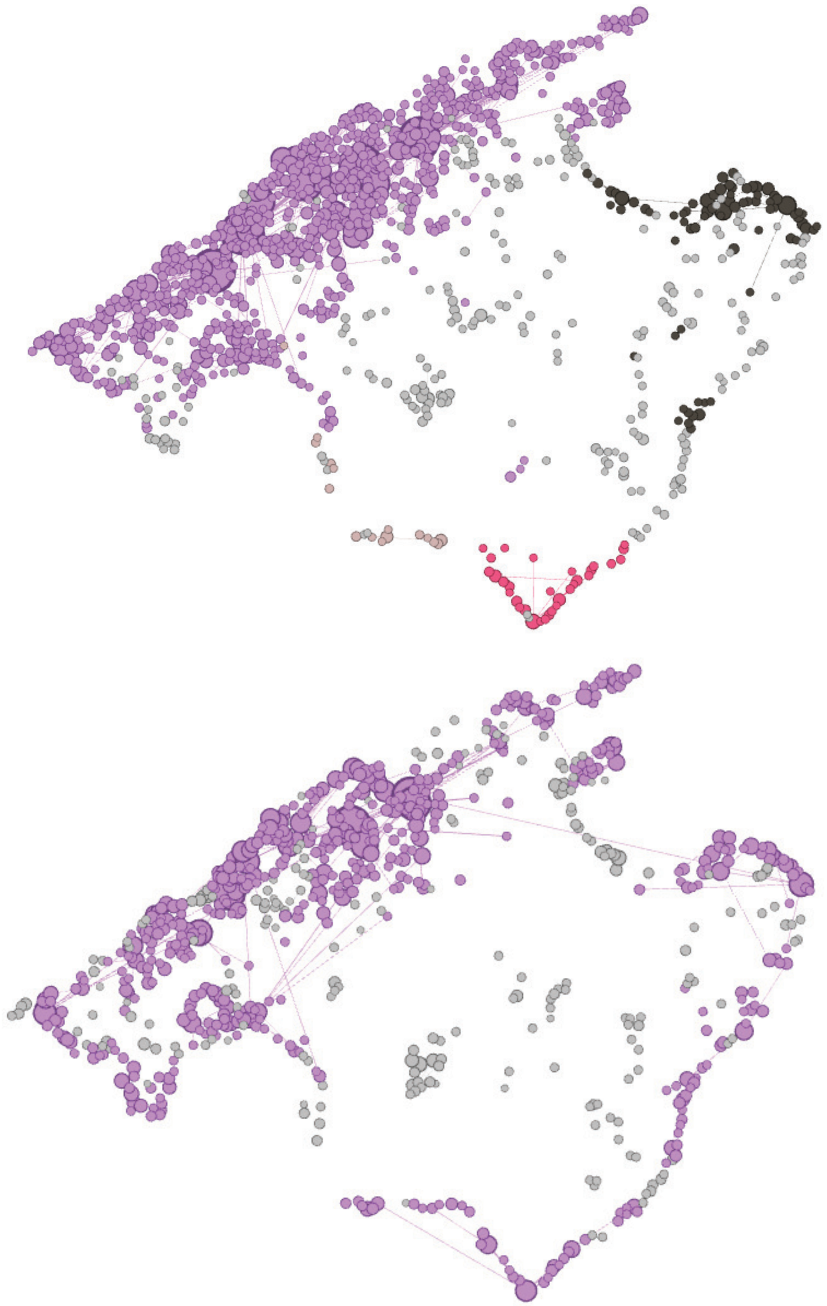

Figure 4. Majorca hiking network. Top: sprint activity. Bottom: summer activity. The size of the nodes represents the number of different hikes from that area. Colours identify different isolated subgrahs.

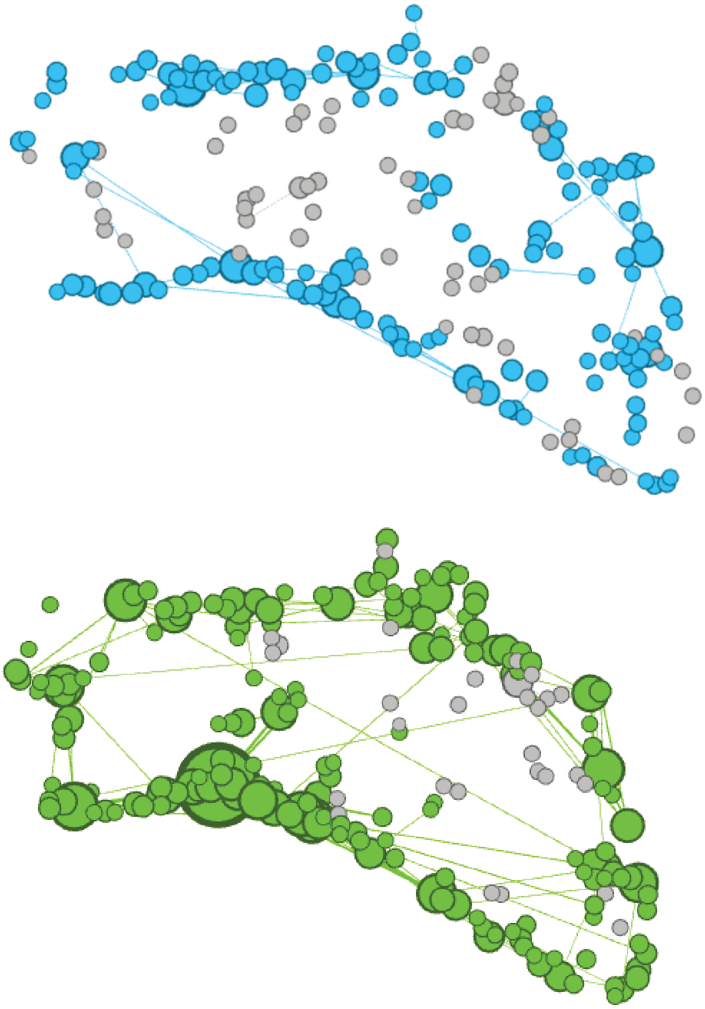

Figure 5. Minorca hiking network. Top: sprint activity. Bottom: summer activity. The size of the nodes represents the number of different hikes from that area. Colours identify different isolated subgrahs.

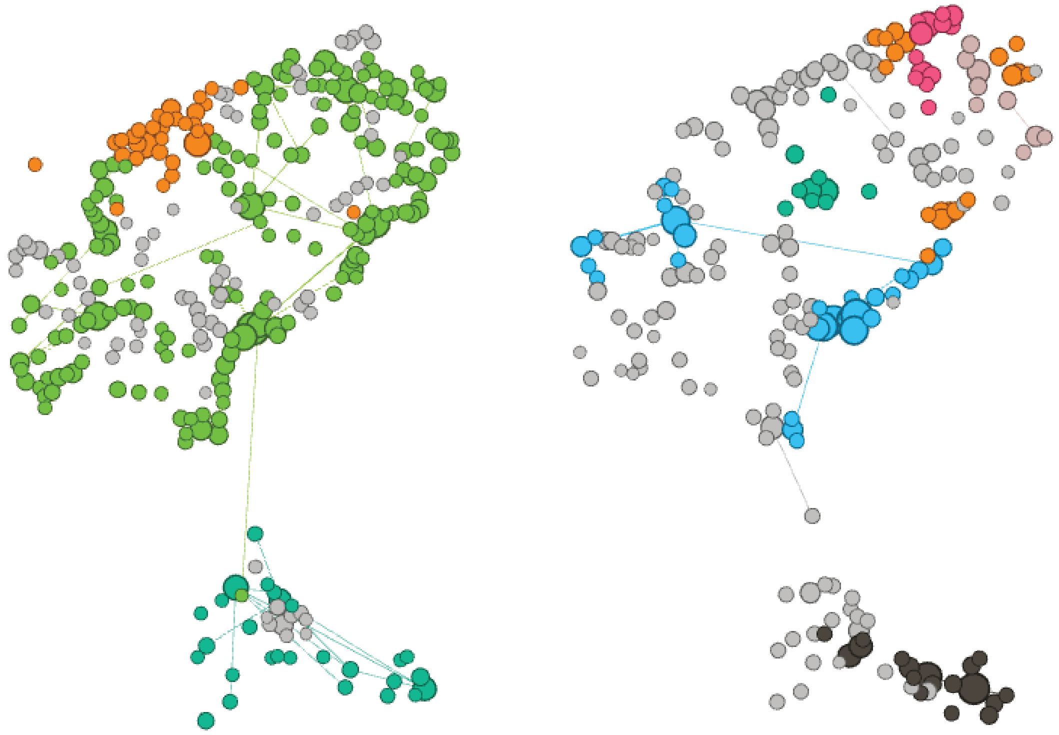

Figure 6. Ibiza & Formentera hiking network. Left: sprint activity. Right: summer activity. The size of the nodes represents the number of different hikes from that area. Colours identify different isolated subgrahs.
